# Mr. Stevenson's Reply to Dr. Farre

**Published:** 1813-03

**Authors:** John Stevenson

**Affiliations:** Great Russell-street


### *To the Editors of the Medical and Physical Journal.*

GENTLEMEN,

THE singular production of Dr. Farre, which appeared in your Journal of last December, under the title of "A Remonstrance to Mr. Stevenson," so fully confirms the truth of what I had previously advanced, that it deserves my acknowledgments, rather than any other animadversion.

There are a few particulars, however, which I cannot forbear noticing. Does Dr. Farre mean to insinuate, that, by *editing only a few most expressive pages* on Cataract, he has enabled surgeons in general to cure that disease; and that *he* has thus destroyed "the partition-wall between the surgeon and oculist?" If the alleviation of human suffering is not less important than the manufactory of a *pin*,* what must we think of Dr. Farre's wish to break down the barriers by which the most experienced and enlightened surgeons of the present day have mutually agreed to a division of labour among themselves?—a division which the public, by common consent, have approved and sanctioned. I shall therefore leave to Dr. F. the task of re-uniting the ophthalmic department with the general practice of surgery. Most heartily do I join with him in regretting that Mr. Saunders has left no manuscript account of his operations for the cataract, excepting "the point" contained in my communication; all the rest of the account must therefore depend upon the accuracy of the compiler's memory.

Having had the good fortune to furnish Dr. Farre with one manuscript, which may serve as a testimony of the author, I shall now afford him another, which may explain the cause of Mr. Saunders's backwardness in appearing before the public. Let Dr. Farre and his friends read the following extract, written *eighteen months* before my esteemed master's lamented death, in *reply* to a letter, in which I urged him *immediately* to publish. Let them read, and lament with every honest man, the hard fate of an enlightened, a modest, and a well-intentioned, improver of science. The following are his words:—"If I had the Reviewers under my thumb, I might possibly introduce my bantling *soon* to the world, as I have now had a great number of cases, and have obtained some fame, but no profit, which might never arrive, if these fellows should deem it proper to damn my performance. I am perfectly aware that an half dozen at

* Vide Dr. Adam Smith on this subject, and an admirable paper among the Essays of Dr. Goldsmith.

least least are on the tiptoe with expectation to do me that especial favor; and, although neither you, nor I, who know a little of the nature of their criticisms, much respect their sentiments, yet the world at large are gulled. What think you of Dr. *****, sitting in an arm-chair, and cutting up Dr. Vetche's Treatise on the Ægyptian Ophthalmia, when he himself had never seen the disease? This is a fact!"

Had Mr. Saunders courted the friendship of Reviewers, instead of other society, he might have dismissed his fears, and, as report says, (with what truth your correspondent perhaps can tell,) either *have reviewed his own work*, or received compliments beyond what his modesty would admit. He might even have enjoyed the satisfaction, if the goodness of his heart had allowed it, of seeing every thing abused which threatened the most distant *competition* with his own writings. On this subject permit me to conclude, with congratulating your readers on the impartiality with which the criticisms in your Journal are conducted.

I am, Gentlemen,
Your obedient Servant,
JOHN STEVENSON.

*Great Russell-street,*
*Jan.* 22, 1813.

